# A safe and simple technique for nasogastric tube insertion in patients with thoracic esophageal cancer surgery

**DOI:** 10.1186/s12957-021-02428-7

**Published:** 2021-11-03

**Authors:** Noriyuki Hirahara, Takeshi Matsubara, Shunsuke Kaji, Yuki Uchida, Tetsu Yamamoto, Ryoji Hyakudomi, Kiyoe Takai, Kazunari Ishitobi, Yoshitsugu Tajima

**Affiliations:** Department of Digestive and General Surgery, Shimane University Faculty of Medicine, 89-1 Enya-cho, Izumo, Shimane 693-8501 Japan

**Keywords:** Nasogastric tube, Esophageal cancer, Esophagectomy

## Abstract

**Background:**

Risk factors for anastomotic leakage include local factors such as excessive tension across anastomosis and increased intraluminal pressure on the gastric conduit; therefore, we consider the placement of a nasogastric tube to be essential in reducing anastomotic leakage. In this study, we devised a safe and simple technique to place an NGT during an end-to-side, automatic circular-stapled esophagogastrostomy.

**Methods:**

First, a 4-0 nylon thread is fixed in the narrow groove between the plastic and metal parts of the tip of the anvil head. After dissecting the esophagus, the tip of the NGT is guided out of the lumen of the cervical esophageal stump. The connecting nylon thread is applied to the anvil head with the tip of the NGT. The anvil head is inserted into the cervical esophageal stump, and a purse-string suture is performed on the esophageal stump to complete the anvil head placement. The main unit of the automated stapler is inserted through the tip of a reconstructed gastric conduit, and the stapler is subsequently fired and an end-to-side esophagogastrostomy is achieved. The main unit of the automated stapler is then pulled out from the gastric conduit, and the NGT comes out with the anvil head from the tip of the reconstructed gastric conduit. Subsequently, the nylon thread is cut. After creating an α-loop with the NGT outside of the lumen, the tip of the NGT is inserted into the gastric conduit along the lesser curvature toward the caudal side. Finally, the inlet of the automated stapler on the tip of the gastric conduit is closed with an automated linear stapler, and the esophagogastrostomy is completed.

**Results:**

We utilized this technique in seven patients who underwent esophagectomy for esophageal cancer; smooth and safe placement of the NGT was accomplished in all cases.

**Conclusion:**

Our technique of NGT placement is simple, safe, and feasible.

## Background

Patients who require nasogastric tube (NGT) insertion for bowel decompression in the context of various pathological conditions are often encountered. NGT insertion following upper gastrointestinal surgery has been reported to be effective in preventing suture failure as a function of intestinal decompression, enhanced detection of postoperative bleeding, and prevention of aspiration pneumonia. However, complications such as misinsertion into the trachea, bleeding upon contact with the anastomotic site, and anastomotic leakage have also been reported with this insertion [1–4]. Anastomotic leakage, recurrent laryngeal nerve palsy, and pneumonia are the three major postoperative complications associated with esophagectomy for esophageal cancer, all of which are sometimes life-threatening. It is well known that the occurrence of these serious complications leads not only to prolonged hospitalization and deterioration of quality of life but also to exacerbation of the long-term prognosis of esophageal cancer [5, 6]. Recent studies have demonstrated that recurrent laryngeal nerve palsy can be avoided by surgical technique or targeted intraoperative monitoring of the nerve [7, 8]. In addition, perioperative, integrated multidisciplinary care of patients can help reduce the risk of developing postoperative pneumonia [9, 10]. Insufficient blood flow and excessive tension across the anastomosis, increased intraluminal pressure of the gastric conduit, structural changes associated with preoperative chemoradiotherapy, and invasive nature of esophageal surgery are contributing factors to the high incidence of anastomotic leakage after esophageal cancer surgery [11–13].

With an aim to maintain low intraluminal pressures in the regions of esophagogastric anastomoses, NGT was inserted for 5 days after esophageal cancer surgery at our institute. Some conditions such as cough, swallowing, and insufficient gastric evacuation increased the pressure on the anastomoses, which may lead to anastomotic leakage. Meanwhile, intraoperative insertion of an NGT after completing an esophagogastrostomy is complicated because it involves a blind maneuver, wherein the tip of the NGT may collide with the anastomosis and cause unexpected mechanical stimulation of the surgical site. Furthermore, upon NGT insertion into the gastric conduit, under endoscopic guidance, following completion of the esophagogastric anastomosis, further mechanical stimulation is exerted on the esophagogastric anastomotic site [14]. In relation to this, our study describes our safe and simple technique developed to place an NGT upon the conduct of an end-to-side, automatic, circular-stapled esophagogastrostomy.

## Methods

At our institution, an end-to-side esophagogastrostomy with a narrow gastric conduit elevated via a posterior mediastinal route was performed at the level of the neck using an automatic circular stapler (CDH, Ethicon, Somerville, NJ, USA) in patients who underwent McKeown’s esophagectomy. In cases wherein an NGT could pass beyond the esophageal tumor, it was inserted into the stomach prior to induction of general anesthesia to prevent mask ventilation-related air retention in the alimentary tract until endotracheal intubation.

**Fig. 1:**
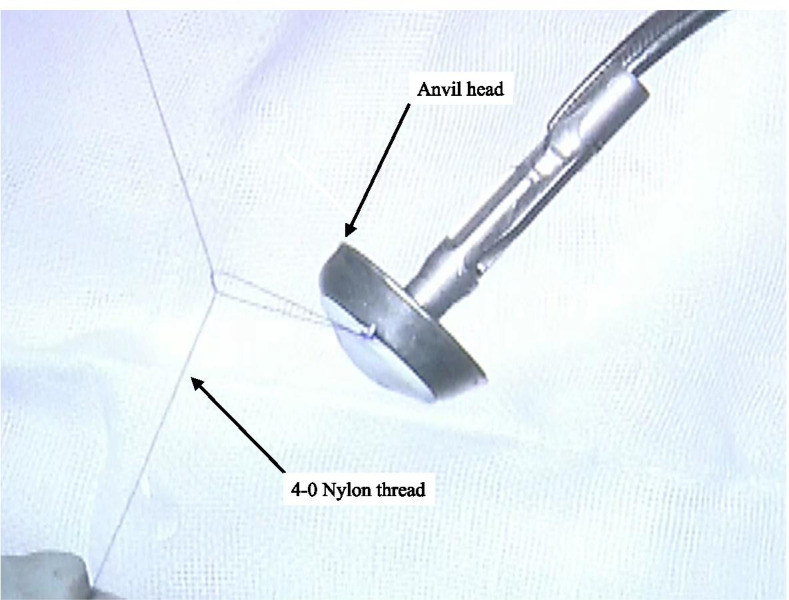
A 4-0 nylon thread is fixed in the narrow groove between the plastic and metal parts of the tip of the anvil head

First, a 4-0 nylon thread was fixed in the narrow groove between the plastic and metal parts of the tip of the anvil head prior to performing an esophagogastric anastomosis (Fig. 1).

**Fig. 2:**
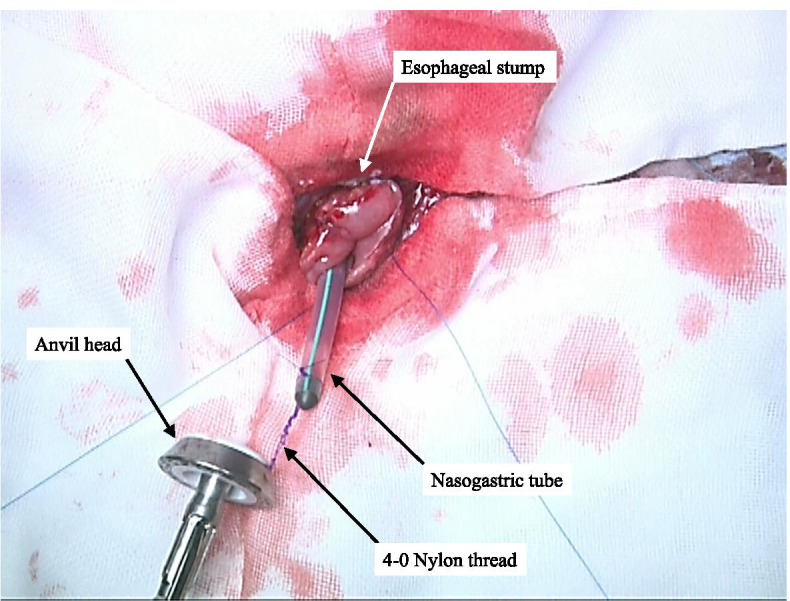
The 4-0 nylon thread applied on the anvil head is connected to the tip of the NGT

**Fig. 3:**
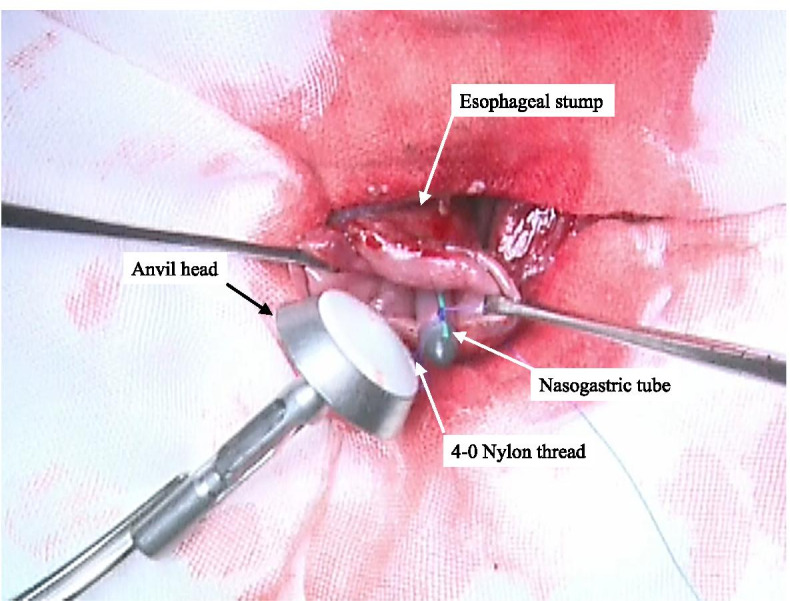
The anvil head is inserted into the cervical esophageal stump as the NGT is slowly and gently pulled out through the nose

**Fig. 4:**
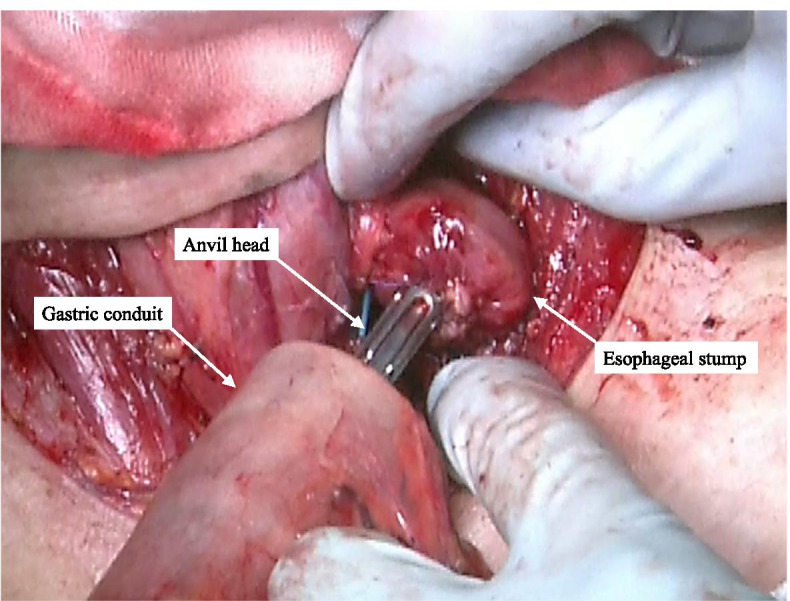
The trocar of the automated stapler is positioned in the planned anastomotic site on the greater curvature of the gastric conduit and joined to the anvil head

**Fig. 5:**
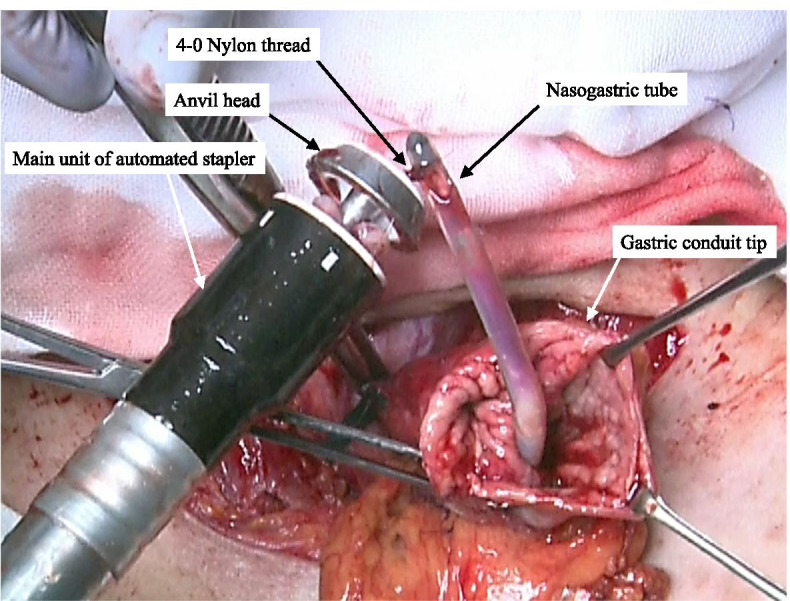
The main unit of the automated stapler is then pulled out from the gastric conduit, and the NGT comes out with the anvil head beyond the esophagogastric anastomosis

**Fig. 6:**
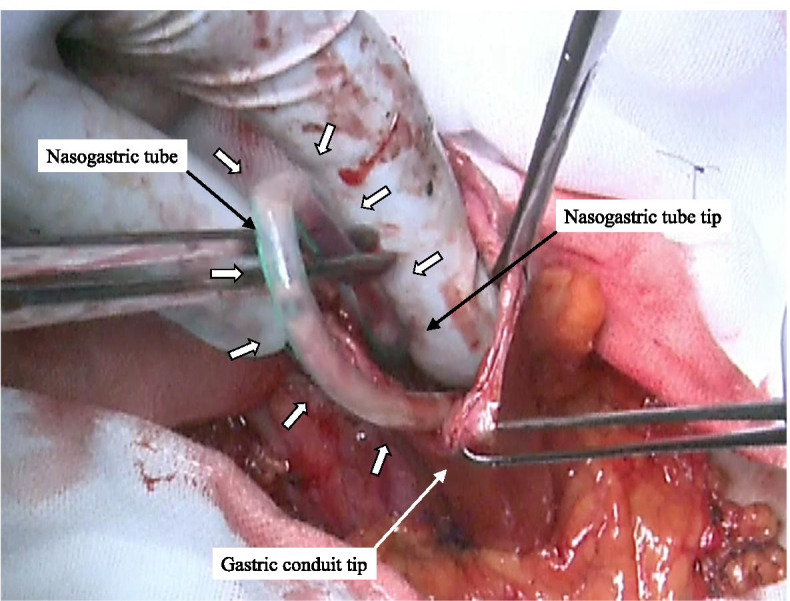
With creating an α-loop (white arrow) with the NGT outside of the lumen, the tip of the NGT is inserted into the gastric conduit along the lesser curvature

When dissecting the esophagus, the tip of the NGT was moved to the oral side away from the dissection line, pushed back, and guided out of the lumen of the cervical esophageal stump that is to be anastomosed. After connecting the nylon thread applied on the anvil head with the tip of the NGT (Fig. 2), the anvil head was inserted into the cervical esophageal stump as the NGT was slowly and gently pulled out through the nose, and a purse-string suture was performed on the esophageal stump to complete the anvil head placement (Fig. 3). The main unit of the automated stapler was inserted through the tip of a reconstructed gastric conduit, and the trocar of the automated stapler was positioned in the planned anastomotic site and then joined to the anvil head (Fig. 4). The stapler was subsequently fired, and an end-to-side esophagogastrostomy was achieved. The main unit of the automated stapler was pulled out from the gastric conduit, and the NGT was removed with the anvil head beyond the anastomosis from the tip of the reconstructed gastric conduit (Fig. 5). Subsequently, the nylon thread was cut. After creating an α-loop with the NGT outside the lumen, the tip of the NGT was inserted into the gastric conduit along the lesser curvature toward the caudal side until the tip of the NGT traverses past the anastomotic segment under either visual or manual guidance (Fig. 6). Once the tip of the NGT had traversed caudally beyond the esophagogastric anastomosis, the NGT has almost no risk of exerting pressure on the anastomosis despite pushing the tube in/out through the nose.

**Fig. 7:**
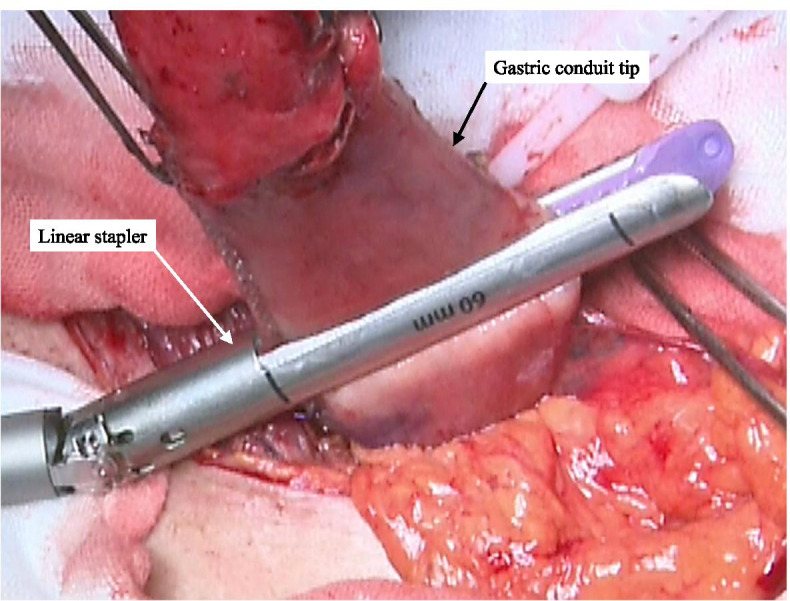
The inlet of the automated stapler on the tip of the gastric conduit is closed with an automated linear stapler

The inlet of the automated stapler on the tip of the gastric conduit was closed with an automated linear stapler (Fig. 7). The anastomosis was re-evaluated in multiple directions, and an air leak test was performed to complete the esophagogastrostomy.

## Results

We used this technique in seven patients (six men and one woman) who underwent esophagectomy for esophageal cancer. All the patients had a preoperative histological diagnosis of esophageal squamous cell carcinoma, and their ages ranged from 61 to 79 years, with a median of 72 years. Five patients presented with a middle thoracic esophageal cancer and two with a lower esophageal cancer.

All the patients underwent McKeown’s esophagectomy. End-to-side esophagogastrostomy with a gastric conduit, which was elevated via the posterior mediastinal route, was performed at the level of the neck using a CDH circular stapler. Smooth and safe placement of the NGT was accomplished in all the patients without any technical difficulty or improper placement. Furthermore, no anastomotic leakage occurred in any patients.

## Discussion

Esophageal cancer surgery is strongly associated with anastomotic leakage compared to other gastrointestinal surgeries. Risk factors for anastomotic leakage include systemic factors, such as increased age, poor nutritional status, and presence of comorbidities; local factors, such as increased intraluminal pressure in the gastrointestinal tract, impaired circulation, and infection; and specific factors associated with highly invasive esophageal surgery. Moreover, it is difficult to keep the anastomosis stable under stress caused due to activities resulting from the jittering motion caused by breathing, cardiac pumping, and swallowing. Circulatory disorders of the reconstructed organs strongly influence the risk of anastomotic leak, which must be addressed and resolved by esophageal surgeons [11–13].

There are two main methods of esophagogastric anastomosis: hand-sewn anastomosis and instrumental anastomosis. The instrumental anastomosis can be divided into end-to-side anastomosis using a circular stapler and end-to-end or side-to-side anastomosis using a linear stapler. We usually perform an end-to-side automatic circular-stapled esophagogastrostomy because it is simple and less susceptible to the influence of the surgeon’s skill level as compared to anastomoses using linear staplers. However, this method results in loss of the gastric wall structure in response to the size of the circular stapler, thereby impairing blood supply in the gastric conduit and resulting in reduced blood flow to the tip of the gastric conduit. In addition, the liner-stapled entry hole for the circular stapler becomes a blind stump, thus presenting a risk factor for anastomotic leakage. This portion is susceptible to the intraluminal pressure arising from the gastric conduit itself [15–17]. Therefore, we believe that NGT placement is essential in minimizing adverse events caused by both the esophagogastric anastomosis and the stump of the gastric conduit, which are associated with increased intragastric pressure from coughing and regurgitation of gastric juices and bile.

The key points of our technique to avoid an unexpected contact with the end-to-side, esophagogastric anastomosis when placing an NGT are as follows: (1) connection of an NGT to the anvil head using a nylon thread, (2) passage of the NGT through the esophagogastric anastomosis in the direction of the thread, (3) guidance of the NGT out of the gastric conduit from its tip and creation of an α-loop with the NGT outside of the gastric conduit, and (4) gentle manual reinsertion of the NGT along the lesser curvature until the tip of the NGT traverses past the anastomotic segment, preventing the gastric tube from returning to the oral side through the esophagogastric anastomosis. At this time, the creation of an α-loop with the NGT outside the gastric conduit enables relatively safer and easier placement of the NGT into the gastric conduit, as shown in Fig. 6.

Although we used the CDH stapler in this study, our technique can be applied using other automatic circular staplers such as the EEA stapler (Covidien, Minneapolis, MN, USA). Three small holes on the anvil head of the EEA stapler can be used to connect to an NGT using a 4-0 nylon thread.

Our study has some potential limitations, including a limited number of specific cases. Moreover, the incidence of anastomotic leakage has not been compared between this technique and the conventional method of blind insertion or insertion under endoscopic guidance. Furthermore, this technique may be used for intrathoracic anastomoses; however, the narrow intercostal space makes it difficult to insert the main unit of the automated stapler into the thoracic cavity. The author has never performed this procedure for intrathoracic anastomosis.

Although the utility of NGT placement in the postoperative management of esophageal surgery is still unclear and controversial [18–20], we hypothesize that decompression of the intragastric conduit using an NGT is helpful in decreasing postoperative complications such as anastomotic leakage and aspiration pneumonia associated with gastroesophageal reflux and delayed gastric emptying. Furthermore, it is useful for detecting these complications. Our technique of NGT placement is simple and safe and may contribute to reducing the associated mechanical forces exerted on the esophagogastric anastomosis and decreasing anastomosis-related complications.

## Conclusions

Our technique of NGT placement during the performance of an end-to-side, automatic, circular-stapled esophagogastrostomy is a safe, simple, and feasible method.

## Data Availability

The datasets used and analyzed during the current study are available from the corresponding author on reasonable request.

## Abbreviation

NGT: Nasogastric tube
